# Implementation status of infection prevention and control and healthcare-associated infection surveillance in Zambian public hospitals: a WHO IPCAF tool assessment

**DOI:** 10.1017/ash.2026.10855

**Published:** 2026-09-21

**Authors:** Steward Mudenda, Joseph Yamweka Chizimu, Joseph Njung’e, Malambo Mutila, Victor Daka, Jimmy Hangoma, Oliver Mweso, Webrod Mufwambi, Clyde Moono Hakayuwa, Martha Mwaba, Ilunga Mutwale, Morreah Champo, Adriano Focus Lubanga, Loveness Sakalimbwe, Nathan Mugenyi, Linda Musonda, Zoran Muhimba, Chileshe Lukwesa, Mpela Chibi, Priscilla Nkonde Gardner, Sandra Diana Mwadetsa, Maisa Kasanga, Zachariah Mupila, Harvey Kamboyi, Sombo Fwoloshi, Evelyn Wesangula, John Bwalya Muma, Nyambe Sinyange, Roma Chilengi

**Affiliations:** 1 Department of Pharmacy, School of Health Sciences, University of Zambia, Lusaka, Zambia; 2 Antimicrobial Resistance Coordinating Committee, Zambia National Public Health Institute, Lusaka, Zambia; 3 Digital Health, Commonwealth Pharmacists Association, Uganda; 4 Department of Digital Systems and Artificial Intelligence (DSAI), Deitaline Technology Limited, Lusaka, Zambia; 5 Department of Public Health, School of Medicine, Copperbelt University, Ndola, Zambia; 6 Department of Pharmacy, School of Health Sciences, Levy Mwanawasa Medical University, Lusaka, Zambia; 7 Department of Clinical Oncology, Cancer Diseases Hospital, Ministry of Health, Lusaka, Zambia; 8 Department of Internal Medicine, Kanyama Level 1 Hospital, Ministry of Health, Lusaka, Zambia; 9 Department of Clinical Services, Kamuzu Central Hospital, Lilongwe, Malawi; 10 Department of Community Health, Faculty of Medicine, Mbarara University of Science and Technology, Mbarara, Uganda; 11 Department of Infectious Diseases, University Teaching Hospitals, Ministry of Health, Lusaka, Zambia; 12 Department of Pathology, University Teaching Hospitals, Ministry of Health, Lusaka, Zambia; 13 Department of Health, World Health Organization, Lusaka, Zambia; 14 Department of Research, School of Medicine, Eden University, Lusaka, Zambia; 15 Pandemic Preparedness, Strengthening Pandemic Preparedness, Eastern and Southern Africa Health Communit, Arusha, Tanzania; 16 Department of Disease Control, School of Veterinary Medicine, University of Zambia, Lusaka, Zambia

## Abstract

**Background:**

Healthcare-associated infections (HAIs) remain a major global public health challenge, with a disproportionately higher burden in low- and middle-income countries (LMICs), including Zambia, where infection prevention and control (IPC) systems face resource and capacity constraints. Effective IPC programs are essential for reducing HAIs, limiting the emergence and spread of antimicrobial resistance (AMR), and improving patient safety. This study assessed the implementation status of IPC programs in Zambian hospitals.

**Materials and methods:**

A descriptive multicenter cross-sectional survey was conducted from June 1–30, 2025, in 18 public hospitals across Zambia. The World Health Organization Infection Prevention and Control Assessment Framework (IPCAF) tool was used to evaluate IPC implementation across eight core components. Descriptive statistics and Welch’s independent-samples t-test were used to compare IPC implementation levels across hospitals.

**Results:**

The overall mean IPC score was 558 out of 800, indicating an intermediate level of implementation. Half of the hospitals (50%, n = 9) achieved advanced implementation, 39% (n = 7) intermediate, and 11% (n = 2) basic levels. Tertiary hospitals performed better than secondary hospitals (mean score 622 vs 507). The highest-scoring components were IPC programs (86%) and guidelines (88%), while the lowest were workload, staffing, and bed occupancy (60%) and IPC education and training (63%). Despite established IPC committees and guidelines, gaps remained in staff training, HAI and AMR surveillance, and infrastructure.

**Conclusion:**

Most Zambian hospitals have achieved intermediate to advanced IPC implementation. However, sustained investment in workforce capacity, surveillance systems, and infrastructure is required to strengthen IPC practices and support national AMR containment efforts.

## Background

Healthcare-associated infections (HAIs) are among the most significant global public health challenges, affecting hundreds of millions of patients annually.^1,2^ They contribute to prolonged hospital stays, increased antimicrobial use (AMU), long-term disability, and substantial economic burdens on healthcare systems.^3,4^ Additionally, HAIs are also among the major causes of preventable mortality globally.^3,5,6^ HAIs are particularly devastating in low- and middle-income countries (LMICs), where the prevalence is estimated (approximately above 17%) to be higher than that of high-income settings due to resource limitations, weak surveillance systems, and inadequate infection prevention infrastructure.^7,8^

Infection Prevention and Control (IPC) is a cornerstone of safe, high-quality, and resilient healthcare systems.^9–11^ It encompasses a range of evidence-based strategies and organizational systems designed to prevent and control the transmission of infections in healthcare settings.^12,13^ Well-implemented IPC programs have proven to substantially lower HAI rates by up to 70%, curb antimicrobial resistance (AMR), and improve patient outcomes.^14–16^ However, HAIs and AMR remain a huge global health issue, especially in LMICs.^6,17^ Middle-income countries bear the highest burden of HAIs, accounting for an estimated 119 million cases annually.^17^ The World Health Organization (WHO) guides on the core components of IPC programs to monitor and strengthen for sustainable IPC systems as key pillars of resilient healthcare systems.^18^

Worldwide, numerous countries have established national IPC programs and integrated them into their overall health security systems.^19^ However, their implementation continues to vary, with significant gaps observed across Africa.^20,21^ The WHO African Region faces unique challenges, including inadequate infrastructure, shortages of IPC-trained personnel, poor access to water, sanitation, and hygiene (WASH) facilities, and limited surveillance of HAIs and AMR.^22,23^ According to WHO assessments, only a few African countries have functional national IPC programs aligned with WHO standards.^19^ Furthermore, healthcare facilities often lack the resources and technical capacity required to sustain IPC activities, leading to high rates of preventable infections and outbreaks of healthcare-associated pathogens such as *Staphylococcus aureus*, *Klebsiella pneumoniae*, and *Acinetobacter baumannii*.^24^

In Zambia, HAIs such as surgical site infections (SSIs) remain a significant yet under-reported public health problem, with some hospitals recording prevalence rates as high as 18%.^25–27^ Alarmingly, these infections are increasingly showing resistance to key antibiotics, thereby contributing to elevated morbidity, mortality, and healthcare costs.^26,27^ Nonetheless, the country has made commendable progress in developing its National Action Plan (NAP) on AMR (2017–2027), which identifies IPC as one of the key strategic objectives for combating AMR. Some Zambian hospitals have designated IPC focal persons, established committees, and functional WASH programs; however, significant gaps persist in training, surveillance, resource availability, and monitoring systems.^11,28,29^ The present assessment aimed to assess the status of IPC programs, identify strengths and weaknesses across the eight IPCAF core components, and guide targeted interventions for improvement.

## Materials and methods

### Study design, site, and population

**Figure 1.**
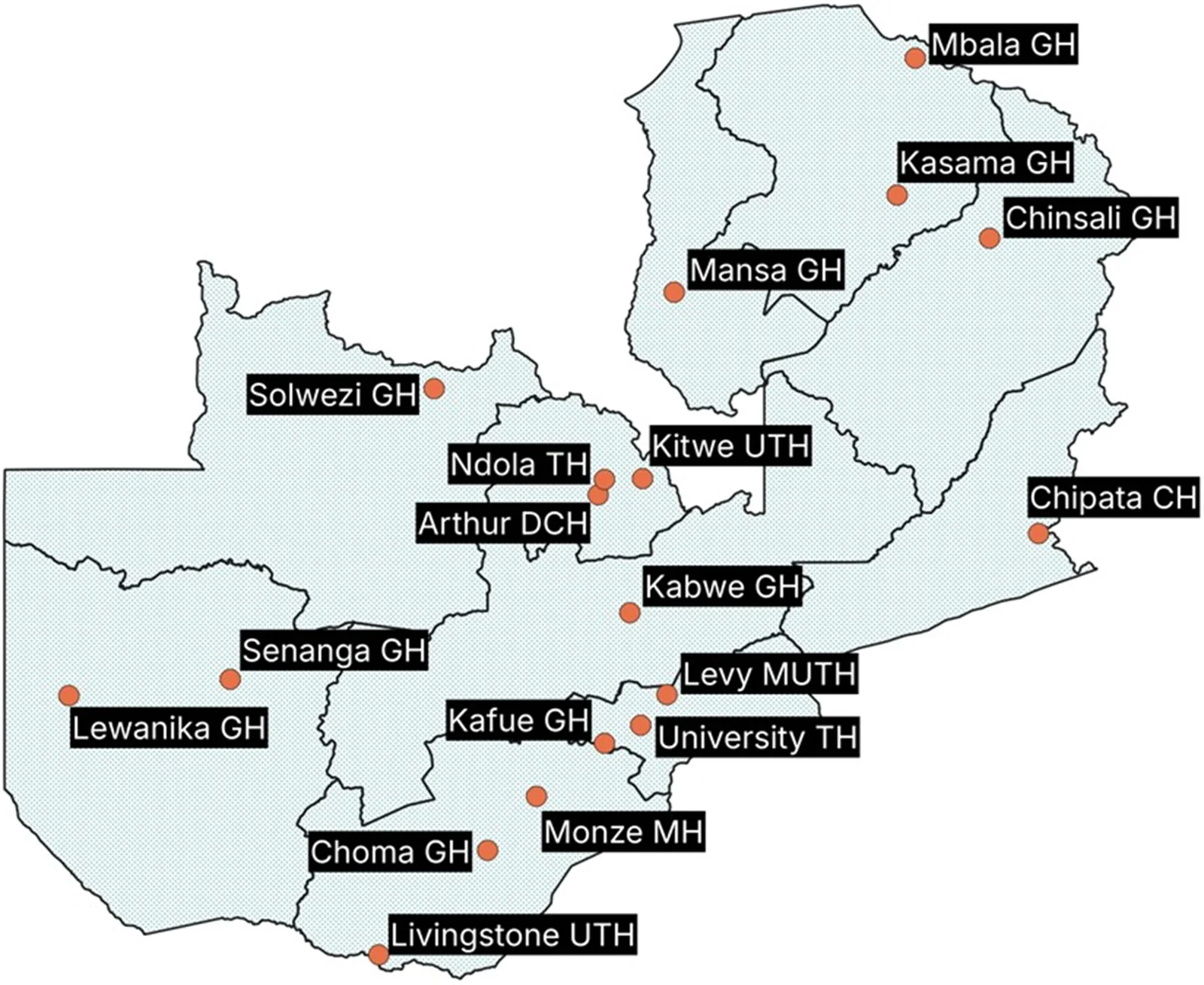
Geographic distribution of selected hospitals included in the IPC assessment in Zambia.

This descriptive, multicentric cross-sectional study was conducted from June 1 to June 30, 2025, to evaluate the status of IPC implementation in selected Zambian hospitals in relation to the WHO minimum requirements for IPC programs. According to the Ministry of Health DHIS2, 2025, Zambia had a total of 11 tertiary hospitals and 25 secondary hospitals during the study period. A total of 18 public hospitals were included, comprising 8 (44%) tertiary-level and 10 (56%) secondary-level institutions. The secondary hospitals included Mansa General Hospital (MGH), Solwezi General Hospital (SoGH), Choma General Hospital (ChoGH), Lewanika General Hospital (LGH), Kafue General Hospital (KafGH), Chinsali General Hospital (CGH), Senanga General Hospital (SGH), Monze General Hospital (MoGH), Mbala General Hospital (MbGH), and Kasama General Hospital (KasGH), while tertiary hospitals included Ndola Teaching Hospital (NTH), Livingstone University Teaching Hospital (LUTH), Levy Mwanawasa University Teaching Hospital (LMUTH), University Teaching Hospital (UTH), Kabwe General Hospital (KGH), Kitwe University Teaching Hospital (KUTH), Chipata Central Hospital (CCH), and Arthur Davison Children’s Hospital (ADCH), as shown in Figure 1.

Hospitals were purposively selected to achieve national geographic representation across Zambia and to include both secondary- and tertiary-level referral hospitals with established IPC committees. The inclusion criteria required hospitals to be publicly owned and managed by the Government of the Republic of Zambia and to have an established IPC Committee. Participants were healthcare professionals directly responsible for planning, coordinating, and implementing IPC activities at their respective hospitals. From each hospital, three key IPC committee members, including the chairperson, secretary, and IPC nurse, were purposively selected to participate, yielding a total of 54 respondents. The three IPC committee members completed the assessment collaboratively through consensus after reviewing supporting documentation. Where discrepancies arose, responses were reconciled through discussion and verification of institutional records, resulting in a single validated IPCAF score for each hospital.

### Data collection

All the data used in this study were collected from June 1 to June 30, 2025, using the WHO IPCAF tool, a globally validated and standardized self-assessment instrument designed to evaluate IPC programs at facility and national levels.^18^ The tool consists of structured questions covering eight core components of IPC implementation. For this study, the original WHO IPCAF questionnaire was applied without any modifications to maintain global comparability, though brief clarifications were provided by the IPC Technical Working Group (TWG) where necessary to ensure contextual understanding during administration.

Eighteen data collectors, comprising medical doctors, pharmacists, nurses, public health professionals, and biomedical scientists, were recruited from the IPC TWG under the Zambian Antimicrobial Resistance Coordinating Committee (AMRCC). All data collectors underwent a three-day intensive training conducted by IPC experts drawn from the AMRCC. The training focused on standardizing data collection procedures, the use of the IPCAF tool, ethical principles, and ensuring data accuracy. Practical sessions on data entry using the Kobo Toolbox electronic platform were also included, with mock entries performed during training to test system functionality and data consistency. The mock entries were removed from the database before the actual data collection.

The data collection teams were organized into six groups of three, each assigned to three hospitals. Data collection involved face-to-face interviews with IPC committee members and document reviews to validate the responses. The IPCAF tool automatically generated scores for each of the eight core components, resulting in a total score ranging from 0 to 800. The scores were categorized as follows: Inadequate (0–200): IPC core components are poorly implemented and require substantial improvement. Basic (201–400): Some elements are in place but require strengthening. Intermediate (401–600): Most components are adequately implemented, though further improvement and sustainability planning are needed. Advanced (601–800): IPC systems are well-established and fully aligned with WHO recommendations.^18^

### Data analysis

All completed questionnaires were reviewed for accuracy and completeness before analysis. Data were analyzed using Python 3.10.11, pandas 2.3.1, and Matplotlib 3.10.3 with reference to the WHO IPCAF tool. Descriptive statistics were generated to summarize the overall and component-specific IPCAF scores, presented as means, standard deviations, frequencies, and percentages. The distribution of hospitals across the four IPC implementation levels (inadequate, basic, intermediate, and advanced) was described. Differences in overall IPCAF scores between secondary (n = 10) and tertiary (n = 8) hospitals were assessed using the Mann–Whitney U test because of the relatively small number of hospitals and the non-parametric nature of the data. Differences in mean overall IPCAF scores between secondary and tertiary hospitals were also assessed using Welch’s independent-samples t-test, which does not assume equal variances. Statistical significance was assessed using two-sided tests with *P* < .05.

### Ethical considerations

The study was approved by the National Health and Research Training Institute (NHRTI) Ethics Committee (Reference No: TRC/C4/09/2023) and conducted in accordance with the Declaration of Helsinki. Written informed consent was obtained from all respondents and facility management before participation. Participants were assured of confidentiality, and all hospitals consented to the publication of anonymized results in scientific outlets.

## Results

**Figure 2.**
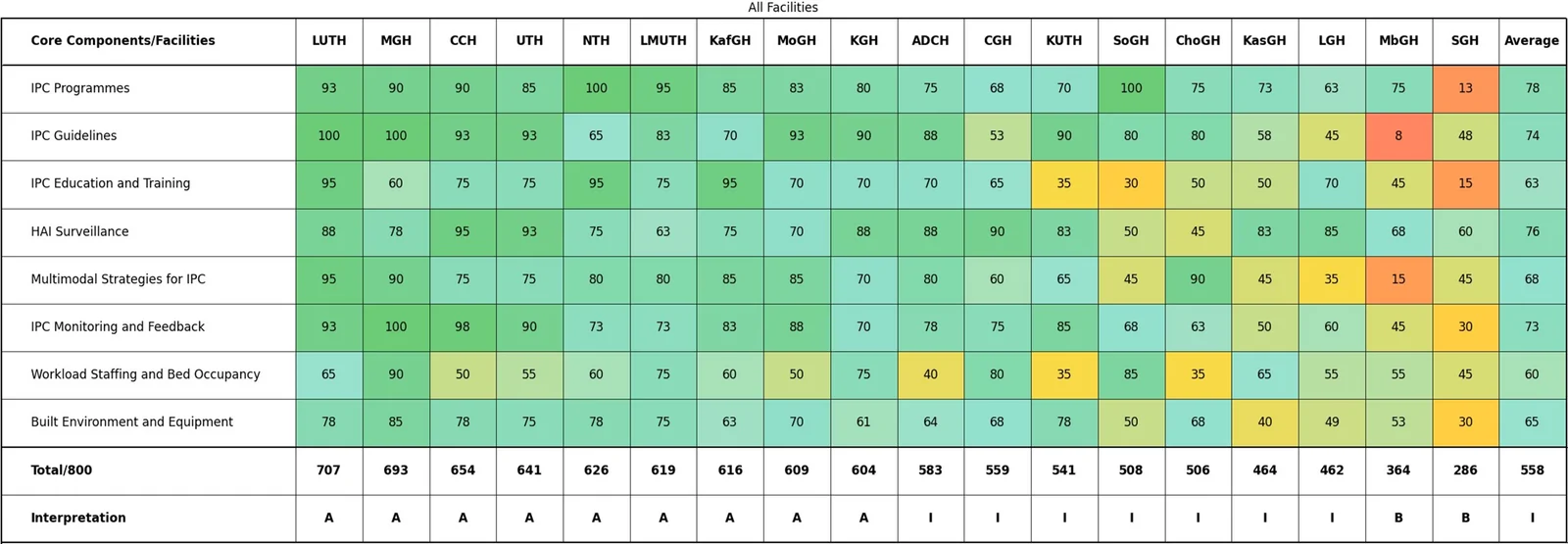
IPC core component score and interpretation level for all participating hospitals (N = 18).

In this study, we included 18 public hospitals and assessed the status of implementation of IPC core components and HAI surveillance. Overall, this study revealed that the implementation of IPC across the 18 public hospitals was intermediate, with an average score of 558 out of 800. The facility-level interpretation categorized nine hospitals as Advanced, seven as Intermediate, and two as Basic. Notably, no hospitals were classified at the inadequate level. Across the core components, IPC Programs (average 78) and HAI Surveillance (average 76) achieved the highest overall mean scores. In contrast, IPC Education and Training (average 63), Multimodal Strategies for IPC (average 68), and Workload Staffing and Bed Occupancy (average 60) were consistently low-performing areas (Figure 2). Substantial differences in IPC implementation were observed across hospitals (Figure 2), with the highest-performing facility scoring more than twice that of the lowest (707 *vs*. 286), *P* = 2.67 × 10^−10^. The strong statistical significance despite relatively small sample sizes (n = 1 per facility) indicates robust and consistent performance patterns within each institution.

Table 1 shows that tertiary hospitals achieved a higher mean score in seven out of the eight core components compared to secondary hospitals, leading to a significantly higher overall IPC score (622 *vs*. 507), as evidenced by the Mann–Whitney: *P* = .021 and Welch’s t-test: *P* = .018. The largest disparities were observed in IPC Guidelines (Tertiary: 88 vs Secondary: 64) and IPC programs (Tertiary: 86 *vs*. Secondary: 72), suggesting tertiary hospitals have more comprehensive and formalized IPC policy frameworks. The smallest gap, where secondary hospitals outperformed tertiary hospitals, was in workload, staffing, and bed occupancy (Secondary: 62 *vs*. Tertiary: 57), indicating relatively greater strain on staffing and bed capacity in the higher-level hospitals (Table 1).

**Table 1.** Comparison of mean IPC core component scores between secondary and tertiary hospitals Table 1 long description.

IPC core component	Secondary hospitals (mean score), n = 10	Tertiary hospitals (mean score), n = 8
1. IPC programs	72	86
2. IPC guidelines	64	88
3. IPC education and Training	55	74
4. HAI surveillance	70	84
5. Multimodal strategies for IPC	60	78
6. IPC monitoring and feedback	66	82
7. Workload staffing and bed occupancy	62	57
8. Built environment and equipment	58	73
Overall IPC score by level of care	507	622

*Note:* IPC, Infection Prevention and Control; HAI, healthcare-associated infections.

**Figure 3.**
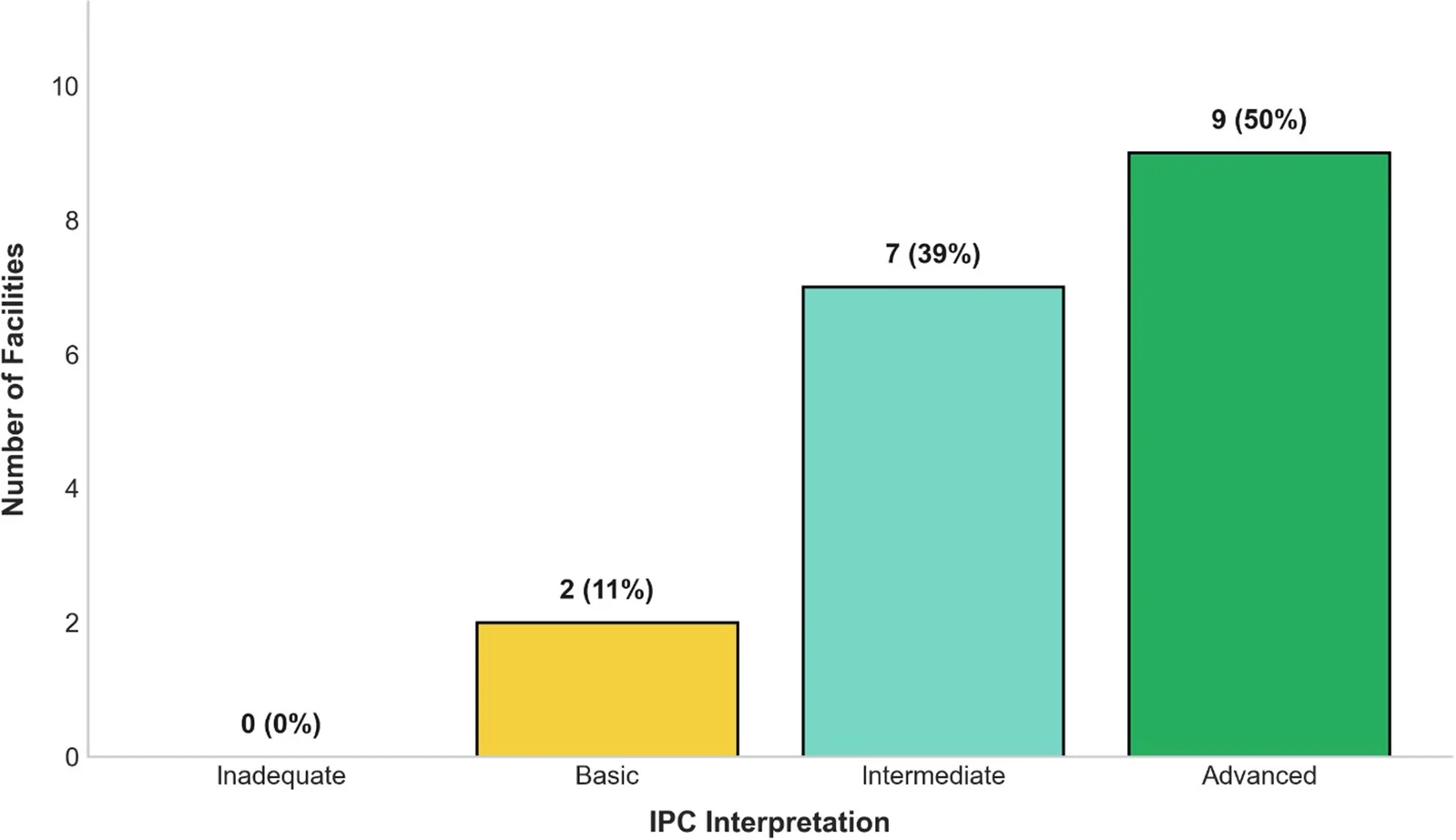
Facility IPC implementation capacity based on IPCAF core component levels (N = 18).

The IPC interpretation capacity data (N = 18) indicate a strong overall proficiency across the surveyed hospitals, suggesting their ability to analyze and understand surveillance findings is robust. The vast majority (89%) demonstrated either Advanced (n = 9, 50%), meaning their IPC core components are fully implemented, or Intermediate (n = 7, 39%) capacity, suggesting most aspects are appropriately implemented, and the focus should be on sustaining and promoting activities. Only a small fraction of hospitals were categorized at the Basic level (n = 2, 11%), where some aspects are in place, but not sufficiently implemented, and none were found to have Inadequate capacity, indicating a high baseline capacity for managing and sustaining IPC programs (Figure 3).

Table 2 shows that the IPC surveillance programs demonstrate strong organizational and methodological foundations, with 88.9% of the hospitals reporting a defined program component, dedicated personnel (100%), trained professionals (88.9%), and adherence to standardized data collection methods (94.4%). However, a critical gap exists in prioritization, as only 50% conduct an exercise to determine local HAI targets, correlating with alarmingly low surveillance of device-associated infections (16.7%). Furthermore, while surveillance data is generally used for improvement (72.2%) and feedback to clinical leaders (94.4%), more than half (55.6%) regularly analyze AMR, and administrative engagement is limited, with only 50% of non-clinical management receiving surveillance feedback (Table 2).

**Table 2.** Assessment of healthcare-associated infection (HAI) surveillance in selected hospitals (N = 18) Table 2 long description.

Question	Answer	n (%)
**Organization of surveillance**		
Is surveillance a defined component of your IPC program?	No	2 (11.1)
	Yes	16 (88.9)
Do you have personnel responsible for surveillance activities?	No	0 (0.0)
	Yes	18 (100)
Have the professionals responsible for surveillance activities been trained in basic epidemiology, surveillance and IPC (that is, capacity to oversee surveillance methods, data management and interpretation)?	No	2 (11.1)
	Yes	16 (88.9)
Do you have informatics/IT support to conduct your surveillance (for example, equipment, mobile technologies, electronic health records)?	No	2 (11.1)
	Yes	16 (88.9)
Do you go through a prioritization exercise to determine the HAIs to be targeted for surveillance according to the local context (that is, identifying infections that are major causes of morbidity and mortality in the facility)?	No	9 (50.0)
	Yes	9 (50.0)
**In your facility, is surveillance conducted for:**		
Surgical site infections?	No	6 (33.3)
	Yes	12 (66.7)
Device-associated infections (for example, catheter-associated urinary tract infections, central line-associated bloodstream infections, peripheral-line associated bloodstream infections, ventilator-associated pneumonia)?	No	15 (83.3)
	Yes	3 (16.7)
Clinically-defined infections (for example, definitions based only on clinical signs or symptoms in the absence of microbiological testing)?	No	4 (22.2)
	Yes	14 (77.8)
Colonization or infections caused by multidrug-resistant pathogens, according to your local epidemiological situation?	No	8 (44.4)
	Yes	10 (55.6)
Local priority epidemic-prone infections (for example, norovirus, influenza, tuberculosis [TB], severe acute respiratory syndrome [SARS], Ebola, Lassa fever)?	No	3 (16.7)
	Yes	15 (83.3)
Infections in vulnerable populations (for example, neonates, intensive care unit, immunocompromised, burn patients)?	No	4 (22.2)
	Yes	14 (77.8)
Infections that may affect health care workers in clinical, laboratory, or other settings (for example, hepatitis B or C, human immunodeficiency virus [HIV], influenza)?	No	6 (33.3)
	Yes	12 (66.7)
Do you regularly evaluate if your surveillance is in line with the current needs and priorities of your facility?	No	3 (16.7)
	Yes	15 (83.3)
**Methods of surveillance**		
Do you use reliable surveillance case definitions (defined numerator and denominator according to international definitions [e.g. CDC NHSN/ECDC] or, if adapted, through an evidence-based adaptation process and expert consultation?	No	2 (11.1)
	Yes	16 (88.9)
Do you use standardized data collection methods (for example, active prospective surveillance) according to international surveillance protocols (for example, CDC NHSN/ECDC) or, if adapted, through an evidence-based adaptation process and expert consultation?	No	1 (5.6)
	Yes	17 (94.4)
Do you have processes in place to regularly review data quality (for example, assessment of case report forms, review of microbiology results, denominator determination, etc.)?	No	2 (11.1)
	Yes	16 (88.9)
Do you have adequate microbiology and laboratory capacity to support surveillance?	No	1 (5.6)
	Yes, can differentiate gram-positive/negative strains, but cannot identify pathogens	0 (0.0)
	Yes, can reliably identify pathogens (for example, isolate identification) promptly	4 (22.2)
	Yes, can reliably identify pathogens and antimicrobial drug resistance patterns (that is, susceptibilities) promptly	13 (72.2)
**Information analysis and dissemination/data use, linkage, and governance**		
Are surveillance data used to make tailored unit/facility-based plans for the improvement of IPC practices?	No	5 (27.8)
	Yes	13 (72.2)
Do you analyze antimicrobial drug resistance on a regular basis (for example, quarterly/half-yearly/annually)?	No	8 (44.4)
	Yes	10 (55.6)
**Do you regularly provide (for example, quarterly/half-yearly/annually) up-to-date surveillance information to?**		
Frontline health care workers (doctors/nurses)?	No	5 (27.8)
	Yes	13 (72.2)
Clinical leaders/heads of department	No	1 (5.6)
	Yes	17 (94.4)
IPC committee	No	2 (11.1)
	Yes	16 (88.9)
Non-clinical management/administration (chief executive officer/chief financial officer)?	No	9 (50.0)
	Yes	9 (50.0)
How do you feedback up-to-date surveillance information? (at least annually)	No feedback	4 (22.2)
	By written/oral information only	6 (33.3)
	By presentation and interactive problem-oriented solution finding	8 (44.4)
Total (N)		18 (100)

*Note:* IPC, Infection Prevention and Control; HAI, healthcare-associated infections; CDC NHSN/ECDC, Centers for Disease Control and Prevention’s National Healthcare Safety Network and the European Centre for Disease Prevention and Control.

## Discussion

This national assessment provides a comprehensive overview of the status of IPC implementation in Zambian public hospitals using the WHO IPCAF tool.

The overall mean IPCAF score of 558/800 indicates that Zambia has achieved considerable progress toward strengthening IPC systems in line with WHO recommendations. The findings demonstrate encouraging progress in establishing IPC structures across both secondary and tertiary hospitals, with most hospitals achieving intermediate to advanced levels of IPC implementation. Similarly, a previous study in Zambia also found that IPC implementation in some Zambian hospitals was at an intermediate level with an average IPCAF score of 594, slightly higher than the current finding.^11^ This finding aligns with similar assessments from other LMICs, where median IPCAF scores often fall within the intermediate range (401–600).^30,31^ The presence of established IPC committees, focal persons, and integration of IPC into quality improvement structures across most hospitals reflects successful national efforts led by the Ministry of Health (MoH) and the AMRCC. These achievements are attributable to recent initiatives, including the NAP on AMR (2017–2027), IPC training programs, and technical support from WHO and partners. Nevertheless, continued efforts are needed in LMICs, including Zambia, in order to achieve high IPC scores in secondary and tertiary hospitals as observed in some high-income countries.^13,32^

In our study, the strongest performance across the IPC core components was seen in the areas of IPC programs, HAI surveillance, and IPC guidelines. This is consistent with earlier research from Zambia, which also reported that IPC programs achieved the highest scores among the eight core components.^11^ These results suggest that many hospitals in Zambia have well-established IPC programs aimed at preventing the development and spread of infections, including HAIs. The present study findings, to some extent, reflect those reported in other LMICs where most hospitals have established IPC programs, developed IPC guidelines, and conduct HAI surveillance, although with some variations.^30,33^

Our study showed that tertiary hospitals had more advanced IPC implementation than secondary hospitals, which generally achieved intermediate IPCAF scores. Tertiary hospitals also demonstrated stronger IPC programs and better implementation of IPC guidelines, likely reflecting greater availability of specialized personnel, infrastructure, and institutional resources. These findings are consistent with those of Kabego et al., who similarly reported higher IPC performance among tertiary healthcare facilities, with a median IPCAF score of 70%.^34^ An intermediate IPCAF classification indicates that most IPC core components are in place but require further strengthening to achieve full compliance. These findings underscore the need for sustained investment in workforce development, surveillance systems, infrastructure, and continuous quality improvement, particularly in secondary hospitals, to reduce disparities in IPC implementation and improve healthcare quality.

Another prominent finding in the present study was the significant disparity in IPC implementation between tertiary (mean IPCAF score 622) and secondary (mean IPCAF score 507) hospitals reflect ongoing health system inequities and technical capacity, as tertiary hospitals have more specialized human resources, better infrastructure investments, and greater access to partner support, whereas secondary hospitals, which often serve large rural populations, face shortages of trained IPC personnel, erratic supply of essential materials, and infrastructural limitations. Secondary-level hospitals, particularly those in remote districts, reported shortages of dedicated isolation spaces, limited laboratory capacity, and frequent stockouts of essential supplies, including disinfectants and personal protective equipment (PPE). This imbalance in capacity across hospitals is consistent with findings from Malawi and Rwanda, where tertiary or central hospitals tend to have higher IPC performance than secondary or peripheral hospitals.^20,30^ Furthermore, this demonstrates that IPC resources must be decentralized to support all the appropriate lower levels of care in each context.^16,35,36^ These findings underscore the need for a tiered national IPC strengthening strategy that prioritizes lower-level hospitals through equitable resource distribution, targeted supervision, and integration of IPC indicators into performance monitoring frameworks. Therefore, deliberate policy efforts should be implemented to ensure that secondary hospitals receive equitable investments in IPC in order for all patients to benefit from standardized IPC prevention measures.

Our study found that the surveillance of HAIs and AMR remains another major gap. Although most hospitals reported collecting infection-related data, few conducted systematic HAI surveillance or analyzed antimicrobial susceptibility trends. Similarly, although most hospitals had established HAI surveillance programs and dedicated surveillance personnel, important gaps remained. Only half of the hospitals prioritized HAIs for surveillance, surveillance of device-associated infections was limited (16.7%), regular AMR data analysis was conducted by only 55.6% of hospitals, and surveillance feedback to non-clinical management was inconsistent. These findings indicate that HAI surveillance systems are established but remain inconsistently implemented, highlighting the need to strengthen surveillance coverage, AMR data utilization, and feedback mechanisms to support evidence-based IPC improvement. This limitation hampers early outbreak detection and evidence-based decision-making. Similar challenges have been observed in other LMICs, where weak surveillance systems and limited microbiology capacity constrain the detection and reporting of HAIs.^37,38^ Consequently, countries can strengthen their HAI surveillance by implementing the protocols as guided by the WHO.^39^ Strengthening laboratory infrastructure, enhancing data management systems, and linking facility-level surveillance with the national AMR surveillance network would be critical to improving infection monitoring and control in Zambia.

Despite the abovementioned progress, the study revealed persistent weaknesses in several core components. Notably, IPC education and training, multimodal strategies, and workload, staffing, and bed occupancy were the lowest-performing areas. The absence of structured, routine IPC training for all healthcare workers undermines the sustainability of infection prevention practices, particularly in hospitals with high staff turnover. These gaps are consistent with findings from studies conducted in Burkina Faso and the DRC, where irregular training and limited mentorship were cited as major barriers to IPC compliance.^33^ To address this, Zambia’s IPC programs need to institutionalize regular capacity-building using the national IPC and AMS curriculum, supported by periodic refresher sessions and practical mentorship.

Infrastructure-related barriers also contribute to inconsistent IPC performance. Inadequate isolation hospitals, overcrowded wards, and unreliable water, sanitation, and hygiene (WASH) systems were common across several hospitals. Such challenges not only increase the risk of HAI transmission but also undermine compliance with hand hygiene and environmental cleaning protocols. Investment in facility infrastructure, WASH improvements, and supply chain resilience must therefore be prioritized to sustain IPC progress and ensure safe healthcare environments. The present study also revealed weaknesses in monitoring, audit, and feedback mechanisms, with limited documentation and the absence of standardized audit tools. Continuous monitoring and feedback are essential for sustaining IPC improvements, as demonstrated in countries where routine auditing led to measurable reductions in HAIs.^40^ Introducing national IPC audit tools and integrating them into existing hospital quality assurance systems could enhance accountability and promote a culture of continuous improvement.

We are aware of the limitations of this study. Its cross-sectional design provides only a snapshot of IPC implementation and cannot establish causality or assess changes over time. Although the WHO IPCAF is a validated assessment tool, it relies partly on self-reported information, which may be subject to reporting bias despite document verification. In addition, the study included only selected public hospitals with established IPC committees, limiting the generalisability of the findings to private and lower-level healthcare facilities. Future longitudinal studies incorporating direct observations and healthcare-associated infection surveillance are needed to evaluate the effectiveness and sustainability of IPC programs.

Overall, IPC structures are in place but require strengthened coordination, regular training, and sustainable resource support for effective implementation (Table 3). Collectively, these findings have important implications for national policy and practice. Zambia’s current progress toward advanced IPC implementation demonstrates a strong foundation upon which to build a resilient, evidence-based IPC program. To accelerate progress, the MoH and partners should strengthen training systems, expand surveillance coverage, improve infrastructure, and ensure a consistent supply of IPC commodities. Moreover, IPC should remain central to the broader One Health approach to AMR, complementing initiatives in AMS, water and sanitation, and infection surveillance.

**Table 3.** Strengths and weaknesses of IPC implementation in selected hospitals in Zambia Table 3 long description.

Aspect	Strengths	Weaknesses
IPC program and governance	Most hospitals have established IPC programs and functional committees. IPC is integrated into facility management and quality improvement systems.	Inconsistent implementation of IPC activities across departments. Some hospitals lack formalized IPC plans and policies.
Training and capacity building	IPC focal persons are appointed in most hospitals. Occasional training is conducted with partner support.	No structured or regular training program for all healthcare workers. Limited refresher courses and induction training on IPC.
Guidelines and policies	Most hospitals possess national or facility-level IPC guidelines.	Incomplete dissemination and limited adherence to guidelines. Lack of regular updates and monitoring of policy implementation.
Surveillance of HAIs and AMR	Some hospitals collect data on infection trends.	HAI surveillance is not systematically implemented. Lack of tools, personnel, and feedback mechanisms for data reporting.
Multimodal strategies	Some hospitals use multimodal approaches in specific areas like hand hygiene.	Weak or absent facilitywide multimodal improvement strategies. Limited monitoring of behavioral and system changes.
Infrastructure and resources	The majority of hospitals have decontamination and sterilization units. Functional waste management and segregation systems.	Inadequate isolation areas and handwashing facilities in some hospital wards. Limited space and infrastructure for IPC activities.
Supplies and equipment	Reliable availability of sterile and disposable items in most hospitals. Availability of essential hand hygiene materials.	Periodic stockouts of disinfectants, PPE, and cleaning materials. Poor maintenance and calibration of sterilization equipment.
Monitoring, audit, and feedback	Some hospitals conduct internal IPC reviews.	Inconsistent monitoring, weak documentation, and a lack of feedback to staff. No standardized audit tools in use.
Staffing and resource allocation	Designated IPC personnel in most hospitals.	Limited dedicated staff exclusively for IPC roles. Inadequate funding for IPC activities.

*Note:* IPC, Infection Prevention and Control; HAI, healthcare-associated infections.

## Conclusion

This assessment provides the first comprehensive national evaluation of IPC implementation in Zambian hospitals since the launch of the AMR National Action Plan. The findings show notable progress toward strengthening IPC structures and practices; however, achieving universal compliance with WHO standards will require sustained investment in workforce training, infrastructure, and surveillance systems. Continued prioritization of IPC is vital to reduce HAIs, curb AMR, and enhance patient safety. Integrating IPC indicators into hospital performance assessments, securing dedicated funding, and promoting multisectoral collaboration will be key to sustaining long-term AMR containment and advancing healthcare quality and safety in Zambia.

## Supporting information

10.1017/ash.2026.10855.sm001 Mudenda et al. supplementary material Mudenda et al. supplementary material

## Data Availability

Data can be made available on request from the corresponding author.

## Supplementary material

The supplementary material for this article can be found at http://doi.org/10.1017/ash.2026.10855.

## Table 1. Long description

A table comparing IPC core component scores between secondary and tertiary hospitals. The table has 9 rows and 3 columns. The columns are labeled IPC core component, Secondary hospitals (mean score), n = 10, and Tertiary hospitals (mean score), n = 8. The rows list the IPC core components and their corresponding mean scores for secondary and tertiary hospitals. Row 1: IPC programs, Secondary hospitals: 72, Tertiary hospitals: 86. Row 2: IPC guidelines, Secondary hospitals: 64, Tertiary hospitals: 88. Row 3: IPC education and Training, Secondary hospitals: 55, Tertiary hospitals: 74. Row 4: HAI surveillance, Secondary hospitals: 70, Tertiary hospitals: 84. Row 5: Multimodal strategies for IPC, Secondary hospitals: 60, Tertiary hospitals: 78. Row 6: IPC monitoring and feedback, Secondary hospitals: 66, Tertiary hospitals: 82. Row 7: Workload staffing and bed occupancy, Secondary hospitals: 62, Tertiary hospitals: 57. Row 8: Built environment and equipment, Secondary hospitals: 58, Tertiary hospitals: 73. Row 9: Overall IPC score by level of care, Secondary hospitals: 507, Tertiary hospitals: 622.

Navigate back to Table 1..

## Table 2. Long description

The table assesses healthcare-associated infection (HAI) surveillance in 18 selected hospitals. It has 20 rows and 6 columns. The columns are labeled Question, Answer, No, and Yes, with sub-columns N and %. The rows detail various aspects of surveillance programs, including organization, personnel, training, data collection methods, and feedback mechanisms. Each row provides the number and percentage of hospitals responding ‘;No’; or ‘;Yes’; to specific questions about their surveillance practices. Notable trends include high percentages of hospitals with defined surveillance programs, dedicated personnel, and trained professionals, but lower percentages in areas like prioritization exercises and surveillance of device-associated infections.

Navigate back to Table 2..

## Table 3. Long description

A table with nine rows and three columns comparing strengths and weaknesses of IPC implementation in hospitals. The columns are labeled Aspect, Strengths, and Weaknesses. The aspects include IPC program and governance, training and capacity building, guidelines and policies, surveillance of HAIs and AMR, multimodal strategies, infrastructure and resources, supplies and equipment, monitoring, audit, and feedback, and staffing and resource allocation. Each row details specific strengths and weaknesses for each aspect.

Navigate back to Table 3..
